# The Roles of Microtubule-Based Transport at Presynaptic Nerve Terminals

**DOI:** 10.3389/fnsyn.2016.00003

**Published:** 2016-02-10

**Authors:** Oleksandr Yagensky, Tahere Kalantary Dehaghi, John Jia En Chua

**Affiliations:** ^1^Research Group Protein Trafficking in Synaptic Development and Function, Department of Neurobiology, Max-Planck-Institute for Biophysical ChemistryGöttingen, Germany; ^2^Interactomics and Intracellular Trafficking Laboratory, Department of Physiology, Yong Loo Lin School of Medicine, National University of Singapore, SingaporeSingapore; ^3^Neurobiology/Ageing Programme, National University of Singapore, SingaporeSingapore

**Keywords:** presynapse, intracellular trafficking, kinesin, Dynein, signaling pathways, synaptogenesis, autophagy

## Abstract

Targeted intracellular movement of presynaptic proteins plays important roles during synapse formation and, later, in the homeostatic maintenance of mature synapses. Movement of these proteins, often as vesicular packages, is mediated by motor complexes travelling along intracellular cytoskeletal networks. Presynaptic protein transport by kinesin motors in particular plays important roles during synaptogenesis to bring newly synthesized proteins to establish nascent synaptic sites. Conversely, movement of proteins away from presynaptic sites by Dynein motors enables synapse-nuclear signaling and allows for synaptic renewal through degradation of unwanted or damaged proteins. Remarkably, recent data has indicated that synaptic and protein trafficking machineries can modulate each other’s functions. Here, we survey the mechanisms involved in moving presynaptic components to and away from synapses and how this process supports presynaptic function.

## Introduction

Neurons possess the capacity to modify their synapses throughout life (Holtmaat and Svoboda, 2009). Being often located significant distances away from the neuronal cell body, biomolecules destined to function at synapses must usually be transported over extended distances before reaching their destinations. Likewise, biomolecules earmarked for degradation or involved in retrograde signaling must be returned to the cell body. Both processes are critically dependent on the intracellular trafficking machinery that is powered by cytoskeletal-based molecular motors. This review will examine the roles of the microtubule-based transport machinery as an integral set of nanomachines supporting the function of presynaptic terminals.

## Molecular Complexity of Synapses and Challenges for Synaptic Trafficking

**Figure 1:**
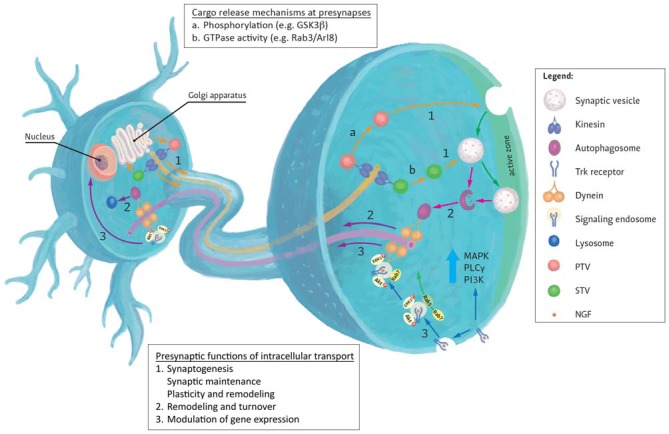
**Generalized representation of trafficking events to and from the presynapse.** (1) Most presynaptic proteins are synthesized in the cell body and transit the Golgi apparatus where they are sorted to specific transporting vesicles (for example, synaptic vesicle precursor transport vesicles (STVs) or Piccolo-Bassoon transport vesicles (PTVs)) and delivered to presynapses by anterograde kinesin motors. Upon reaching their destinations, several mechanisms are known to mediate cargo unloading including: (a) post-translational modifications (e.g., phosphorylation) of kinesin motors, adaptors and cargoes or (b) via the action of GTPases and their effectors. Released cargoes can then be used to build new presynaptic sites (synaptogenesis), maintain existing synapses or contribute to synaptic plasticity. (2) Degradation and turnover of excess or damaged proteins by the autophagy pathway also contributes to presynaptic homeostatic maintenance. Autophagosomes formed here are returned to the cell body by retrograde Dynein motors. (3) Retrograde transport can also be used to convey extracellular signals received at presynaptic terminals to the nucleus where they modulate gene expression. These signals impact the growth and survival of neurons and mediate their response to injury. An example of such responses can be evoked by the binding of neurotrophins (for example NGF) to presynaptic Trk receptors. Activation of these receptors evokes local signaling response at presynapse. Alternatively, receptor-ligand complexes are endocytosed and form signaling endosomes containing other activated signaling molecules. These endosomes are then transported by Dynein to the cell body to induce changes in gene expression or directly regulate signaling cascades. Sizes of molecules and vesicles are not drawn to scale.

Presynaptic terminals play critical roles in mediating neurotransmitter release during synaptic transmission (Figure 1). To fulfill these roles, mature terminals possess hundreds of different proteins that are intricately assembled into molecular nanomachines involved in various aspects of presynaptic function (Morciano et al., 2009; Südhof, 2012; Boyken et al., 2013; Wilhelm et al., 2014). In particular, synaptic vesicles (SVs) and active zones (AZs) are key components involved in this process. Elegant proteomic analysis of SV composition by Takamori et al. (2006) demonstrated that, despite its small size, SVs exhibit remarkable complexity and diversity in their protein content. Proteins directly involved in vesicle exocytosis such as the SNARE proteins (syntaxins, synaptobrevins and SNAPs), the calcium sensor synaptotagmin, V-ATPase and vesicular neurotransmitter transporters (VGLUT and VGAT) were all detected. Similarly, AZs that demarcate sites of neurotransmitter release by recruiting SVs and voltage-gated Ca^2+^ channels also comprise of a large ensemble of proteins including AZ proteins such as RIM, RIM-BP, Munc13, ELKS/CAST/ERC and α-liprin that participate in SV docking and priming (Südhof, 2012; Chua, 2014). While this complexity and diversity of proteins in the presynapse has long been appreciated, we are only starting to unravel the principles underlying the movement of these cargoes to and away from these sites.

## Microtubule-Based Motors as Nanomachines to Transport Presynaptic Cargoes

Diffusion can be effective for migration of small cargoes within very short distances (Encalada and Goldstein, 2014). However, the process becomes highly inefficient with increasing cargo size and distance to be travelled. The substantial gap separating most synapses and the neuronal cell body necessitates the employment of the intracellular trafficking machinery to shuttle presynaptic proteins between these sites. In general, Kinesin and Dynein motor protein families are involved in delivering and removing vesicular content to and from presynaptic sites, respectively (Hirokawa et al., 2010; Kevenaar and Hoogenraad, 2015). Both motor families utilize microtubule cytoskeletal networks to move cargo. Microtubules are inherently polar and the plus ends of microtubule tracks point away from the cell body in axons (Kapitein and Hoogenraad, 2011). Thus, in axonal transport, Kinesin motors are primarily responsible for moving cargoes from sites of synthesis to presynaptic sites, while Dynein motors mediate movement of biomolecules from synapses towards the cell body. In comparison, Myosin motors are generally regarded to be involved in short-range actin-based transport and will not be covered here (Cingolani and Goda, 2008; Hirokawa et al., 2010).

## Anterograde Transport to the Presynapse Involves Kinesin Motors

Many synaptic proteins traffic through the Golgi apparatus en route to synapses after their synthesis in the cell body (Figure 1; Sytnyk et al., 2004; Sann et al., 2009). Kinesin motors play important roles not only during the Golgi export of these proteins but also during subsequent trafficking to their destinations (Akhmanova and Hammer, 2010). Presynaptic proteins transported by Kinesin motors include SV and AZ proteins. The precise mechanisms governing the sorting and loading of cargoes onto their respective motors remain unclear but phosphorylation of cargoes, adapters and motors has been implicated as an important regulatory step in this process (Sato-Yoshitake et al., 1992; Lee and Hollenbeck, 1995; Morfini et al., 2002; Chua et al., 2012).

The trafficking of two broad categories of presynaptic cargoes has been studied. Synaptic vesicle precursor transport vesicles (STVs) carrying SV proteins were shown to be delivered to presynaptic sites by Kinesin-3 motors (KIF1A and KIF1Bβ; Yonekawa et al., 1998; Ahmari et al., 2000; Zhao et al., 2001; Sabo et al., 2006). Loss of KIF1A and KIF1Bβ in neurons caused significant reductions in SVs and their proteins in presynaptic terminals. On the other hand, vesicular transport of presynaptic membrane proteins, such as SNAP-25 and syntaxin-1, are mediated by Kinesin-1 motors (Diefenbach et al., 2002; Su et al., 2004; Chua et al., 2012). AZ proteins including Bassoon, Piccolo and RIM are also co-transported with SNAP-25 and synataxin-1 as Piccolo-Bassoon transport vesicles (PTVs; Shapira et al., 2003; Cai et al., 2007). Interestingly, emerging evidence suggests that presynaptic cargoes are probably not as discretely grouped and an appreciable cargo overlap during transport does exist (Goldstein et al., 2008; Maas et al., 2012; Wu et al., 2013).

## Delivering Cargoes to Specific Synapses

Site-specific release of cargoes is one of the least understood steps in trafficking of presynaptic components. Given that an average mammalian neuron possesses thousands of synapses (Beaulieu and Colonnier, 1985), the transport machinery has to discriminate between the varying needs of different presynaptic sites and deliver the appropriate cargoes to these sites accordingly. The simplest explanation could be that cargoes are released upon reaching the ends of microtubule tracks at the presynapse (Rizzoli, 2014). These cargoes would be then redistributed between neighboring presynaptic sites by diffusion, retrograde transport on the same microtubule track or other mechanism (Darcy et al., 2006; Opazo et al., 2010; Staras et al., 2010). Nevertheless, release of cargo caused exclusively by discontinuity of microtubules cannot fully explain specificity of targeted delivery of presynaptic proteins. Local activation of signaling cascades involved in the arrest of anterograde transport can contribute to this process. One such pathway relates to the small GTPase Rab3 which is a marker of mature SVs and is co-transported with STVs to the presynapse (Fischer von Mollard et al., 1990; Schlüter et al., 2004). DENN/MADD is a Rab3 guanine nucleotide exchange factor (GEF) previously shown to regulate neurotransmitter release (Yamaguchi et al., 2002). DENN/MADD was shown to bind both KIF1A and KIF1Bβ and promote anterograde transport of vesicles associated with Rab3 in the GTP-bound state (Niwa et al., 2008). Either locking Rab3 in the GTP-bound state or deletion of the DENN domain containing the putative enzymatic GEF activity interferes with the transport of these vesicles to distal axons. These results suggest that local factors that induce GTP hydrolysis and cycling of Rab3 between its nucleotide bound states could act as a trigger to release cargoes at distal presynaptic sites.

Phosphorylation of motors and cargoes has been implicated in synaptic cargo transport (Sato-Yoshitake et al., 1992; Vagnoni et al., 2011; Chua et al., 2012). Similarly, phosphorylation-dephosphorylation cycles of these proteins are also likely to affect synapse specific delivery of cargoes. One possible candidate for this is GSK3β. Phosphorylation of kinesin light chains by GSK3β does not affect the binding of kinesin to microtubules but instead releases membrane cargoes from the motor (Morfini et al., 2004). GSK3β is a general inhibitor of anterograde trafficking but is selectively active in growth cones (Morfini et al., 2004; Polleux and Snider, 2010; Weaver et al., 2013). Local activation GSK3β can therefore contribute to the release of kinesin cargoes and formation of synapses *de novo* along the leading edge of growing axon.

During synaptogenesis, both PTVs and STVs move bi-directionally along axonal processes and coordinated trafficking of a portion of STVs and PTVs to specific sites along axons has been documented (Ahmari et al., 2000; Shapira et al., 2003; Sabo et al., 2006; Bury and Sabo, 2011). Pausing of STVs and PTVs coincide with cargo deposition in response to a hierarchy of cues from synaptogenic molecules (such as trans-synaptic adhesion molecules) and initiates the formation of nascent presynaptic sites (Lucido et al., 2009; Siddiqui and Craig, 2011; Chia et al., 2013). Coordination between the small GTPase Arl8 and the Mitogen-activated protein (MAP) kinase signaling pathway has been implicated in facilitating site-specific unloading of these cargoes to developing presynaptic sites in *C. elegans* (Wu et al., 2013). In this regard, Arl8 has been proposed to prevent undesirable premature assembly of presynaptic proteins during axonal transport while signaling through JKK-1/JNK-1 promotes capturing and integration of these proteins to the presynapse. It remains to be determined if Arl8 also serves a similar function during synaptogenesis in mammalian neurons.

In addition to the involvement of specific signaling pathways, recent studies suggest that some aspects of synaptic delivery could be inherently stochastic in nature. In mature synapses, a fraction of recycling SVs is distributed between mature synapses—a phenomenon that occurs in the scale of minutes (Darcy et al., 2006). Remarkably, this exchange occurs not only between neighboring boutons but also between remote synapses. Activity-dependent regulation of synapsin-1 phosphorylation at position Ser-551 by Cdk5 modulates clustering and dispersion of SVs between different presynaptic sites (Verstegen et al., 2014). This would indicate that redistribution of some synaptic components can occur following their delivery to synapses by Kinesin motors. In *Drosophila*, dense core vesicles destined for presynaptic terminals are initially observed to oscillate between proximal and distal axonal regions, with movement mediated by both Kinesin and Dynein motors (Wong et al., 2012). While undergoing retrograde movement to the neuronal soma, a portion of these vesicles becomes recruited to presynaptic terminals along the axons. Remarkably, these retrograde moving vesicles are preferentially captured by distal as compared to proximal presynaptic sites. Similar observations have also been made for SV precursor transport vesicles in *C. elegans* (Maeder et al., 2014). Whether or not an undefined mechanism is responsible for mediating the gradient decline in synaptic capture remains to be determined.

## Retrograde Trafficking is Mediated by Dynein

In addition to delivering cargoes, transport of biomolecules away from presynaptic sites towards the cell body also plays important regulatory as well as homeostatic roles for presynaptic function (Figure 1). Retrograde movement of specific messengers in response to synaptic activity can trigger transcription of genes in the nucleus whose products contribute to long-term modifications of synaptic behavior or as a response to injury (Panayotis et al., 2015). Furthermore, sustained and optimum presynaptic function requires removal and degradation of worn out or damaged presynaptic proteins or organelles for turnover and degradation (Bezprozvanny and Hiesinger, 2013). In both instances, Dynein mediates the retrograde movement of these cargoes (Hirokawa et al., 2010; Roberts et al., 2013).

## Synapse-Nuclear Signaling

Communication between synaptic termini and cell body is vital for modulation of synaptogenesis, long-term potentiation, axonal growth and responses to mechanical damage (Panayotis et al., 2015). Synapse-to-soma signaling can be divided into fast response component, that involves propagation of Ca^2+^ ion waves through neurites as a signal, and slow response component, which utilizes retrograde transport of signaling molecules to the cell body. Ca^2+^ signaling from the postsynapse to soma is well studied and has been demonstrated to be involved in modulation of gene expression (Bading et al., 1993; Greer and Greenberg, 2008; Hagenston and Bading, 2011). This method of signaling may also play a significant role following injury (Ziv and Spira, 1995; Wolf et al., 2001) and during axon growth (Yamane et al., 2012). Calcium waves can be propagated to soma, for example, through axon-specific ER-like structures (Merianda et al., 2009), or locally induce other signaling pathways (Ghosh-Roy et al., 2010) to evoke responses in the cell body.

In comparison to the fast response, which is most efficient for synapses located closer to the cell body, the slow signaling component involves retrograde transport of activated signaling molecules (for example kinases or receptors) to the nucleus using Dynein motors (Schmieg et al., 2014; Panayotis et al., 2015). Among these, the best studied are the neurotrophins, a group of extracellular signaling proteins including NGF, BDNF and NT-3/4 (Huang and Reichardt, 2001). At the presynapse, binding of neurotrophins to their receptors (either tropomyosin receptor kinase (Trk) family or p75^NTR^ receptors) induces their autophosphorylation and activates downstream signaling pathways through MAPK, PLCγ and PI3K (Delcroix et al., 2003; Howe and Mobley, 2005; Schmieg et al., 2014). Interestingly, activation of Trk receptors depends on the type of neurotrophin and synaptic terminus it is applied to Kuruvilla et al. (2004). Stimulation of axonal terminus by NT-3 in sympathetic neurons promotes axonal growth by local activation of TrkA independent of TrkA internalization. In contrast, NGF-mediated activation of TrkA induces its endocytosis and co-transport with downstream signaling molecules pERK1/2 and pAkt to the soma (Kuruvilla et al., 2004). Multiple signaling molecules can be transported to the soma on common vesicles. This phenomenon led to the hypothesis of the signaling endosome (Schmieg et al., 2014), a dynamic organelle that co-transports numerous signaling molecules from the axon periphery to the cell body. Transporting multiple signaling molecules on a single vesicle fulfills two functions. Firstly, it potentially allows interactions to occur between components of the different signaling pathways carried by the vesicle that can result in either enhancement or inhibition of the eventual response. Second, vesicular transport of kinases or receptors preserves them in the active, often phosphorylated, state along the way. A large amount of data demonstrates that interruption of Dynein-mediated retrograde transport of neurotrophins leads to ablation of their signaling (Purves, 1976; Salehi et al., 2006; Sharma et al., 2010). For instance, disruption of BDNF-induced TrkB signaling by perturbing snapin-Dynein interaction, which prevents their retrograde transport to the nucleus, was recently shown to reduce dendritic outgrowth (Zhou et al., 2012). Signaling endosomes are assumed to carry a range of signaling molecules and markers that define its fate during transport to the soma. In agreement with this, early endosomal markers Rab5 and EEA1 colocalize with TrkA at distal tips of neurites (Howe et al., 2001; Delcroix et al., 2003). Upon maturation, signaling endosomes exchange Rab5 for Rab7, which is a late endosome marker. This event is crucial for targeting TrkA to the cell body as overexpression of dominant negative Rab7 in PC12 cells disrupts transport of neurotrophin receptors complexes (Deinhardt et al., 2006).

In the cell body, signals derived from presynaptic sites are converted into specific responses, such as changes in gene expression. For instance, the downstream target of NGF, ERK5 can activate transcription factor MEF2D, which in turn induces expression of anti-apoptotic protein bcl-w, promoting survival of the developing sensory neurons (Pazyra-Murphy et al., 2009). Transcription factors can also be transported to nucleus directly from distal axonal regions. In support of this, several studies indicate that local translation of importins, proteins indispensable for nuclear transport of cytoplasmic proteins, can take place in injured axons (Hanz et al., 2003; Yudin et al., 2008; Perry et al., 2012). Knockout of importin β1 in axons led to a substantial change in the transcriptome of neurons inflicted with axonal injury (Perry et al., 2012). Studies of retrograde signaling from postsynapse revealed that importins bind to transcription factors and mediate their transport to the soma using Dynein motors (Meffert et al., 2003).

## Synapse Homeostasis and Turnover of Presynaptic Proteins

Presynaptic proteins are subjected to turnover during homeostatic and activity-dependent plasticity (Lazarevic et al., 2011; Cohen et al., 2013). Apart from proteasome-dependent degradation, autophagic degradation of presynaptic components is emerging as an important strategy to deal with unwanted biomolecules (Yamamoto and Yue, 2014; Menzies et al., 2015). Presynaptic components targeted for degradation by autophagy include SVs and α-synuclein (Friedman et al., 2012; Binotti et al., 2015). However, details concerning how these components are directed to sites of degradation remain unclear. Efficient clearance of autophagosomes is challenging in neurons since many of these vesicles are formed at the distal axons located far from lysosomes that are largely present in the soma (Yamamoto and Yue, 2014; Menzies et al., 2015). Retrograde transport of autophagosomes is an essential step towards the fusion of both types of vesicles thereby enabling completion of the degradation process. An important question is to understand how neurons employ particular sorting mechanisms to achieve this.

A recent study reported that retrograde movement of autophagosomes at distal sites in neuronal axons involves binding of JIP1, a Kinesin-1 activator, to LC3 present on autophagosomes (Verhey and Hammond, 2009; Fu et al., 2014). This interaction prevents JIP1 from activating Kinesin-1. Moreover, JIP1 recruits the Dynein motor complex by binding p150(Glued), a component of the dynactin complex which is an integral Dynein subunit shown to be important for initiating retrograde trafficking from synaptic sites (Allan, 2011; Lloyd et al., 2012; Fu and Holzbaur, 2013). Intriguingly, abrogation of JIP1 binding to LC3 by deleting the LC3-interaction region on JIP1 inhibits fusion of the autophagosomes with lysosomes—an essential step towards the eventual degradation of autophagosomal content. Because this deletion also impairs retrograde trafficking of autophagosomes, it remains to be clarified if the inhibition is related to the transport defect or if indeed that JIP1 does participate in a yet understood role in the autophagy pathway.

An alternative route for the recruitment of Dynein to autophagosomes appears to be facilitated by snapin. Previous data indicated that a complex of Dynein and snapin resides in late endosomes and is responsible for driving their retrograde transport (Cai et al., 2010). A proportion of autophagosomes was observed to fuse with late endosomes to form amphisomes, a known intermediate step in the autophagic pathway (Lamb et al., 2013; Yamamoto and Yue, 2014; Cheng et al., 2015). In doing so, these autophagosomes acquire the ability to move in a retrograde fashion via the Dynein-snapin complex present on late endosomes. Thus, the study provides additional insights into trafficking routes taken by autophagsomes en route for degradation.

## Part of a Team: Cooperation Between Kinesin and Dynein Motors in Moving Presynaptic Proteins

Although protein transport has been conventionally viewed to be unidirectional, Kinesin and Dynein motors are known to bind simultaneously to most synaptic cargoes (Encalada and Goldstein, 2014). Simultaneous binding of cargoes to motors of opposing directionality confers several advantages. First of all, retrograde movement of degenerated synaptic components or endocytosed material would be only possible if Dynein is available at the distal parts of the axon. This would necessitate Dynein travelling with cargoes in anterograde direction to enable distal localization (Yamada et al., 2010). Secondly, microtubule tracks along axons are not contiguous. Microtubule breaks or clogging of the axon can happen along the path of cargo transport and switching between motors of opposite polarity may allow the cargo to bypass the hindrance. Finally, many studies indicate that opposite-polarity motors activate one another and such an interaction is essential for efficient cargo transport in either direction (Barkus et al., 2008; Ally et al., 2009; Uchida et al., 2009). Nevertheless, it remains unclear what determines which motor becomes dominant in a given environment. Specific regulatory mechanisms are likely to determine the directionality of cargo movement. For instance, adaptor protein Huntingtin (Htt) together with huntingtin-associated protein-1 (HAP1) links BDNF-containing vesicles to Dynein motor. Upon IGF signaling and consequent activation of the Akt pathway, Htt becomes phosphorylated at serine 421 which in turn recruits KHC to the cargo and favors its anterograde transport (Colin et al., 2008).

## Presynaptic Plasticity and intracellular Trafficking

Synapses undergo constant remodeling (plasticity changes) in accordance to their history of activity which is a basis for learning and memory (Holtmaat and Svoboda, 2009). Such remodeling can occur as subtle changes in synaptic composition or overt modifications to synaptic morphology (Lazarevic et al., 2011; Weyhersmüller et al., 2011). Recent studies indicate that in addition to constitutive transport to synapses, the trafficking machinery can itself also respond to changes in synaptic activity and contribute to synaptic plasticity and remodeling. In particular, activity-enhanced trafficking of ionotropic glutamate receptors to post-synaptic density contributes to synaptic plasticity (Yin et al., 2011; Hoerndli et al., 2013, 2015).

Similarly, activity-mediated bidirectional modulation of synaptic behavior and trafficking has also been observed at the presynapse. Repetitive stimulation of cultured neurons increases formation of new presynaptic terminals that is dependent on trafficking of presynaptic components by Kinesin-1 (Cai et al., 2007). Likewise, higher levels of the Kinesin-3 motor KIF1A corresponding with increased trafficking of presynaptic cargoes by Kinesin-3 motors has also been observed in mice placed in an enriched environment in a learning paradigm (Kondo et al., 2012). Direct morphological changes have also been described. Prolonged activation of photoreceptor synapses causes loss of T-bars at presynaptic sites—a phenomenon associated with the removal of AZ proteins liprin-α, Bruchpilot by the Kinesin-3 motor Imac (Sugie et al., 2015).

## Future Perspectives

In the fast changing environment of synaptic communication, neurons must cope with the varying demands of different synapses. Emerging evidence indicates that the intracellular trafficking machinery does not only respond passively to replenish or remove biomolecules from synapses but can also pro-actively respond to the needs of individual synapses. Key questions that remain to be clarified include: (1) What mechanisms govern the loading of cargoes to their motors and, upon reaching their destinations, unloading the cargoes; (2) How heterogeneous is the composition of different cargo subsets and is the composition of these subsets regulated over the course of the lifespan of the neuron; and (3) What are the mechanisms regulating specificity of targeting to different synapses? Noteworthy, impairment of intracellular trafficking has been identified in a range of neurodegenerative disorders such as Alzheimer’s and ALS where synapse loss presents itself as an early feature during disease onset (Millecamps and Julien, 2013). A better understanding of mechanisms governing the specificity of targeting to different synapses and the precise contribution of synaptic protein trafficking to synapse health will be important questions to address. Recent advances in optogenetics and super-resolution microscopy techniques, coupled with characterization of the molecular identities of cargoes carried by different motors will be important to bring us one step closer towards unraveling these intriguing questions (Ballister et al., 2015; Hell et al., 2015; van Bergeijk et al., 2015).

## Author Contributions

The manuscript was conceived by JJEC and written by JJEC, OY and TKD. OY and TKD contributed equally to the work.

## Conflict of Interest Statement

The authors declare that the research was conducted in the absence of any commercial or financial relationships that could be construed as a potential conflict of interest.
